# Atherosclerosis: From Molecular Biology to Therapeutic Perspective 3.0

**DOI:** 10.3390/ijms24086897

**Published:** 2023-04-07

**Authors:** Ida Perrotta

**Affiliations:** Department of Biology, Ecology and Earth Sciences, Centre for Microscopy and Microanalysis, University of Calabria, Arcavacata di Rende, 87036 Cosenza, Italy; idaperrotta@yahoo.it

Atherosclerosis is a multifactorial chronic disease triggered and sustained by different risk factors such as dyslipidemia, hypertension, diabetes mellitus (DM), smoke, elevated homocysteine, and hormones [1]. Atherosclerosis develops over many years with a long asymptomatic phase and progresses through a complex interplay between vascular cells, infiltrating inflammatory cells, growth factors/cytokines, oxidative stress, and lipids. In the early stage of lesion formation, low-density lipoprotein (LDL) particles accumulate in the intimal layer of the artery where they undergo oxidative modifications, thus acquiring proinflammatory and immune-stimulatory properties. With time, histologic changes have become increasingly complex, and these initial lesions (called fatty streaks) are evolving toward fibroatheromas which are composed of a lipid-rich atheromatous core covered by a fibrous cap with a loosely arranged matrix dominated by collagen fibers, smooth muscle cells (SMCs), and inflammatory cells. Progression to major acute cardiovascular events (i.e., acute myocardial infarction, sudden cardiac death, and stroke) is often triggered by an atherosclerotic plaque complicated by calcification, endothelial erosion, intraplaque hemorrhage, and, finally, rupture. In recent years, the rapid development of a vast array of techniques in the field of cellular and molecular biology has led to a more in-depth understanding of the molecular history of atherosclerosis and the cellular phenotypes that occur during vascular remodeling. These include biological molecules, biochemical pathways, and processes that, together with the migration, proliferation, and apoptosis of vascular cells, are vital to the pathogenesis of atherosclerosis [2,3,4].

This Special Issue entitled “Atherosclerosis: From Molecular Biology to Therapeutic Perspective 3.0” contains a combination of original articles and reviews written by an international panel of researchers and reviewed by several other experts in the field of cardiovascular sciences, focused on the molecular mechanisms related to the pathogenesis of the disease, the methods that can detect and characterize the lesions, and the potential novel therapeutic strategies for targeting atherosclerotic plaques.

SMCs, as the major cell component of the vessel wall, play an integral role in vasoconstriction and blood pressure regulation and maintain vascular structural integrity. SMCs can also participate in vascular remodeling in both physiological or pathological states by virtue of their capacity to change their phenotype in response to changes in local environmental cues. Under healthy conditions, they exist in a contractile, well-differentiated phenotype. In cases of disease or injury, SMCs undergo a profound transformation to a less differentiated state, acquiring a proliferative potential and migratory properties. A wide range of regulatory factors and signaling pathways can promote this phenotypic transition [5,6]. Miyabe and colleagues demonstrated that treatment of human aortic SMCs with porphyromonas-gingivalis-derived LPS leads to the phosphorylation of p38 MAPK and SAPK/JNK via TLR4/MyD88 and produces an increase in cell proliferation, while the TLR4-mediated activation of ERK in a MyD88-independent pathway culminates in the enhancement of both proliferation and migration. These results provide new insights to explain the potential role for periodontal pathogens in the induction of atherosclerosis.

As briefly mentioned earlier, ox-LDLs are thought to play a central role in the pathogenesis of atherosclerosis. OxLDL has been demonstrated to promote monocyte migration into the vessel wall, endothelial cell (ECs) activation and dysfunction, foam cell formation, SMC migration and proliferation, and to exert powerful pro-inflammatory and pro-oxidative stress effects [7]. Additionally, in the context of cancer biology, increased levels of oxLDL have been found in patients with prostate, colorectal, breast, ovarian, and lung cancer, and animal research has clearly shown the impact of a fat-enriched diet on prostate, colon, and liver cancer development and progression [8,9,10,11]. In this Special Issue, the mechanisms of action of oxLDL have been investigated in relation to head and neck tumorigenesis. Scalia et al. have developed an original method to produce oxLDL and demonstrate that the oxLDL exposure of head and neck cancer cells with a human papilloma virus negative status promotes lipid uptake, upregulates the expression of CD36 and LOX-1 scavenger receptors, and decreases their propensity to migrate.

The interaction of LDL particles with the arterial glycosaminoglycan (GAG) chain(s) present in the extracellular matrix and on cell membranes significantly contributes to the focal retention of lipids, increases the oxidation levels of LDL in the intima, and induces the formation of foam cells during atherogenesis [12]. Recently, Kamato et al. demonstrated that thrombin, via its receptor PAR-1, transactivates both the EGFR and TGFBR1 to stimulate proteoglycan synthesis and the elongation of GAG chains on proteoglycans, consequently increasing lipid binding [13]. In this Special Issue, the same group confirm and extend previous findings, suggesting that the transactivation of EGFR and TGFBR1 by PAR1 specifically occur via Gαq-dependent pathways. These results might open new avenues to develop pharmaceutical interventions aiming to prevent early lipid deposition within the vessel wall.

Vascular inflammation plays critical roles in all phases of the atherosclerotic process, from its beginning to the onset of clinical complications [14]. Perticone et al. have analyzed the expression profiles of different immune-mediated inflammatory markers [Toll-like receptor (TLR) 2, TLR4, nuclear factor kβ, interleukin (IL)-1β, IL-6, IL-8, IL-10, and tumor necrosis factor] in newly diagnosed hypertensive patients with and without 1 h post-load hyperglycemia and demonstrated that the group of patients characterized by a worse glucometabolic profile also possessed a greater activation of the innate immune system. The data presented in this research article help to clarify the pathophysiological role of the immune system and the inflammatory cascade within the arterial wall, and they identify a different risk profile in the hypertensive population with abnormalities of glucose metabolism.

According to the assumption that a wide range of cells of both innate and adaptive immunity are relevant to atherosclerosis initiation and progression [15], Saigusa et al. investigated the impact of cardiovascular disease, DM, and sex on CD4+ T cells, while Eligini et al. discussed the central roles of macrophages in the development of atherosclerotic plaques and examined the different techniques currently used in proteomics that can led to a better understanding of the molecular biology of atherosclerosis, its progression, behavior, and treatment.

Growing evidence suggests that vascular inflammation is associated with adventitial and perivascular vascularization. Vasa vasorum (VV) provide nourishment to the media and facilitate the transport of inflammatory cells from the perivascular adipose tissue to the adventitia [16,17]. Within the adventitial/perivascular area, clusters of MPO+ macrophages produce and release angiogenic growth factors that can act both in a paracrine fashion to support the existing vessels but, in association with cardiovascular risk factors, can also induce the formation of new blood vessels and the expansion of MPO+ clusters, leading to the desynchronization and deregulation of angiogenic and immune responses. Bogdanov and colleagues demonstrated that the rates of (neo)vascularization directly correlates with the extent of intimal hyperplasia and inflammation in an animal model of arterial injury and with pre-implantation stenosis in CABG (coronary artery bypass graft) surgery conduits. The authors concluded that these pathological processes are initiated by damage involving the lumen surface of VV.

The contribution of inflammation to atherosclerosis has been also analyzed in the review by Richter and colleagues, who discussed novel pro-inflammatory and pro-atherosclerotic risk factors that are closely related to systemic lupus erythematosus (SLE) inflammation, and which determine an increased risk for the occurrence of early cardiovascular events.

Dabravolski et al. summarized recent data demonstrating the biological role of pericytes in various aspects of atherosclerosis pathogenesis and discussed how novel molecular and cellular insights into pericyte biology may help to find new candidate targets for more specific and effective therapeutic modalities.

An interesting correlation between plasma fortilin levels and the severity of coronary artery disease (CAD) has been proposed by Aoyama and co-workers, while the review by Gardin et al. has summarized the various methods currently employed for exosome isolation, with a focus on platelet-derived exosomes, and the authors have discussed the role these extracellular vesicles play in the pathogenesis of atherosclerosis and their therapeutic possibilities.

Recently, obstructive sleep apnea (OSA) has emerged as an independent risk factor for atherosclerosis. OSA may contribute to atherosclerosis via direct and indirect mechanisms. Endothelial dysfunction, sympathetic stimulation, and the generation of pro-inflammatory cytokines induced by OSA may play a crucial part in the pathogenesis of atherosclerosis [18,19]. Clinical and experimental studies also showed that OSA can promote an inappropriate activation of the aldosterone/MR (mineralocorticoid receptor) system. In this regard, it is well-established that MR activation in the cardiovascular system is able to promote hypertension, fibrosis, and inflammation [20]. The paper by Badran and colleagues has provided an in-depth exploration of the relationship between OSA and MR activation, its possible deleterious effects on atherosclerosis development, and the potential clinical benefits that could be derived from blocking the MR.

In recent years, numerous studies have highlighted the role of the miR-17/92 cluster in cardiovascular physiology, and dysregulation in the expression levels of miR17-92 cluster members has been broadly implicated in cardiomyopathy, aberrant cardiomyocyte differentiation, myocardial infarction, and cardiac aging [21,22]. miR17-92 expression is known to be modulated by dietary compounds, especially by extra-virgin olive oil and beer [23,24]. On the basis of these previous data, Díez-Ricote et al. investigate whether trimethylamine-N-oxide (TMAO), a gut microbiota-derived metabolite produced from dietary nutrients, possesses the ability to modulate the expression of miR17-92 cluster members in two different cellular models (macrophages and hepatocytes). The authors demonstrate that TMAO can upregulate some members of the miR-17/92 cluster (i.e., miR-17 and miR-92a) and can increase the expression of SERPINE1 and IL-12, at the gene and protein levels, thus promoting the development of inflammation and atherosclerosis.

Kamato and colleagues perform an extensive review of the various non-mouse animal models of atherosclerotic plaque formation and rupture (including hamsters, rats, rabbits, zebrafish, pigs, and non-human primates), with the aim to provide a basis to select the most relevant model to investigate the molecular mechanisms underlying the disease process and its clinical consequences.

Finally, Romero-Cabrera et al. reviewed the available evidence on the ability of novel lipoprotein biomarkers to accurately predict CVD risk among middle-aged adults without history, signs, and symptoms of known CVD, while Vellasamy et al. has explained how the aging of the immune system (immunosenescence) contributes to atherosclerosis.

I would like to express my special appreciation and thanks to all editorial board members, reviewers, and authors; without their cooperation, the present Special Issue would not have come into being.

I sincerely hope that you will enjoy reading this Special Issue and that it will stimulate new ideas for improvement. 

Happy reading!
